# Effect of irreversible electroporation parameters and the presence of a metal stent on the electric field line pattern

**DOI:** 10.1038/s41598-020-70308-3

**Published:** 2020-08-11

**Authors:** Annemiek M. Hogenes, Cornelis H. Slump, Gerben A. te Riet o.g. Scholten, Martijn R. Meijerink, Jurgen J. Fütterer, Cornelis J. H. M. van Laarhoven, Christiaan G. Overduin, Martijn W. J. Stommel

**Affiliations:** 1 Department of Radiology, Nuclear Medicine and Anatomy, Radboud University Medical Centre, P.O. box 9101 (766), 6500 HB Nijmegen, The Netherlands; 2 Department of Robotics and Mechatronics, University of Twente, Enschede, The Netherlands; 3 Department of Radiology and Nuclear Medicine, Amsterdam University Medical Centre, Vrije Universiteit-Cancer Centre Amsterdam, Amsterdam, The Netherlands; 4 Department of Surgery, Radboud University Medical Centre, Nijmegen, The Netherlands

**Keywords:** Surgical oncology, Cancer therapy, Translational research, Cancer, Biomedical engineering

## Abstract

The final ablation zone created with irreversible electroporation (IRE) depends on the size, shape and strength of the electric field that is influenced by several parameters. A profound understanding of the effect of IRE parameter alterations on the electric field are a prerequisite for a safe and effective treatment. Here, we demonstrate a semolina in castor oil model that enables visualization of the static electric field developed by a high-voltage generator between two needle-electrodes. We intuitively visualize the variation in electric field line pattern for selected IRE parameters; active needle length, inter-needle distance, applied voltage and presence of a nearby metal stent, by cameras in three dimensions. The observations were compared to and supported by two-dimensional numerical simulations of the electric field. Our semolina model visualizes the disturbance of the electric field by a metal stent, potentially leading to an incomplete tumour ablation between the needles. The reduction in electric field strength and the area at risk for incomplete tumour ablation are confirmed by the numerical simulations. The semolina model provides insight in the fundamental physics of the electric field, the effect of alterations in IRE parameter combinations and presence of a metal stent within the ablation zone.

## Introduction

Irreversible electroporation (IRE) is theoretically a non-thermal ablation technique, which makes it especially suitable for ablation of tumours near vital structures, such as locally advanced pancreatic tumours^1–3^. IRE uses a high-voltage external electric field and electric pulses of microsecond duration to change the transmembrane potential of tumour cells, resulting in permanent permeabilization of the cell membrane^4–6^. Membrane permeabilization disturbs the cell’s mechanisms to maintain homeostasis, finally leading to cell death via a necrotic pathway presumably or apoptosis^4,7–9^. Tumour cells are ablated while the extracellular matrix, collagen and elastic fibres, such as blood vessels and bile ducts remain intact^9,10^.

Biliary drainage is frequently required in patients with pancreatic tumours. The use of a metal stent is usually preferred because of fewer stent-related complications (e.g. cholangitis) and less stent dislocations compared to plastic stents^11,12^. It is currently unknown whether IRE can be safely and effectively applied in patients with a metal stent in situ close to the ablation site. Detrimental effects of a metal object on IRE outcomes have been suggested in literature. In a case report, severe complications have been described after IRE treatment in the proximity of a metal stent, potentially caused by unintended thermal effects^13^. A retrospective clinical study demonstrated that IRE near small metal surgical clips (< 1 cm to tumour margin or needle electrodes) resulted in distortion of the electric field and inadequate coverage of the tumour, a less effective cell death and reduced treatment efficacy^14^. The presence of a metal clip was also significantly related to local tumour progression. In vivo experiments in swine and pig liver showed a redistributed and unpredictable ablation zone and thermal damage due to the proximity of a metal clip or stent^15,16^. Contradictory results with regard to temperature effects, electric field disturbance, and safe performance of IRE without complications in proximity of metal implants or stent were shown in ex vivo, in vivo and simulation studies^17–20^. The oncologic outcome of IRE is potentially influenced by a metal stent. But when and to what extent a metal stent affects the electric field distribution and associated thermal effects, both in terms of efficacy and safety, remains unclear^15,20^. Thermal effects without the presence of a metal stent have been observed in clinical practice as well, especially directly around the needle-electrodes^21–23^. An inhomogeneous electric field distribution and corresponding locally high current density gives rise to these thermal effects^22,23^. Thermal effects are difficult to predict, with potential consequences for local control of IRE procedures and damage to adjacent critical structures.

The size and shape of the ablation zone depends on several IRE parameters, such as the pulse timing, applied voltage, electrode configuration and active needle length as well as tissue properties^15,24–27^. Visual insight in the electric field line pattern may offer a more profound understanding of the influence of IRE parameter settings, locations of potential thermal effects, and presence of a nearby metal stent on the ablation zone. This could enable the determination of parameter settings to provide a precise and controlled tissue ablation. The aim of this study was to visualize the variation in electric field line pattern for selected IRE ablation parameters and the presence of a metal stent in proximity of the needles.

**Figure 1:**
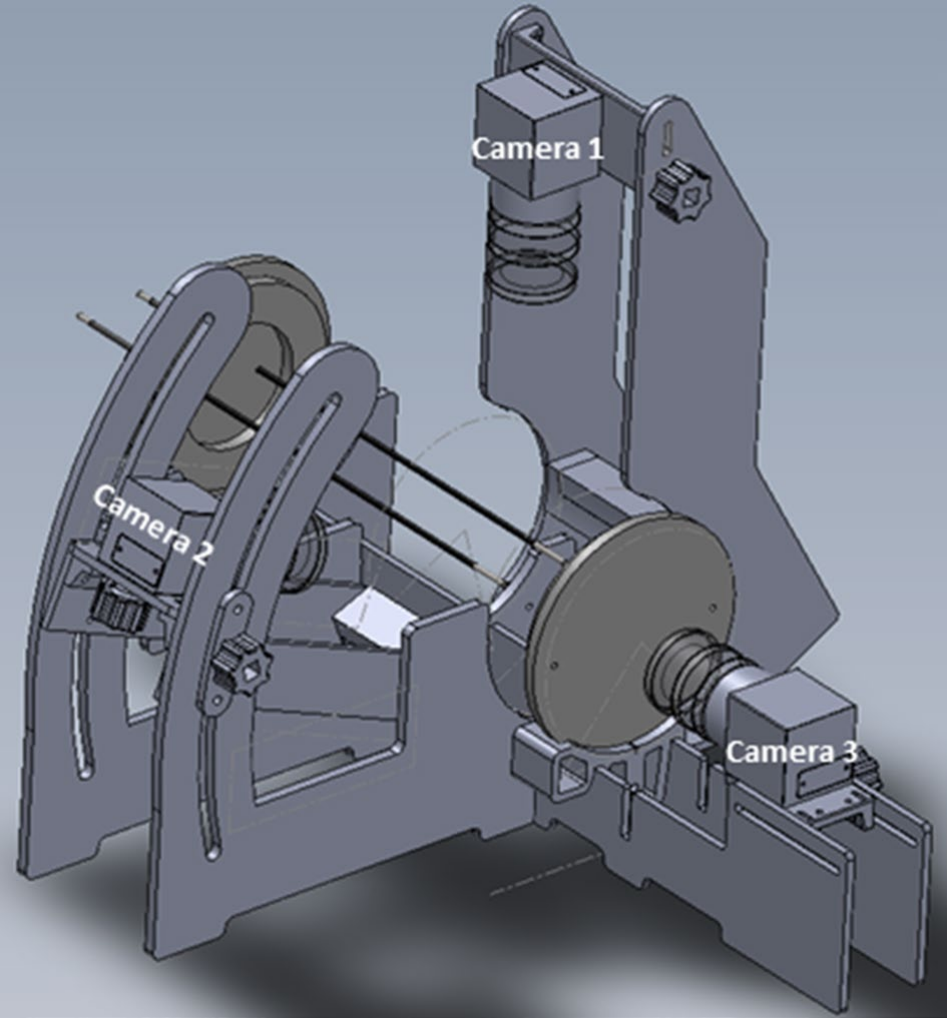
Experimental setup. As reference, the needle-electrodes have an active needle length of 1.5 cm and inter-needle distance of 2.5 cm. Camera 1 visualizes the semolina pattern in the frontal plane, camera 2 in the longitudinal plane and camera 3 in the transverse plane. SolidWorks (SolidWorks, version 2019, www.solidworks.com, Waltham, MA, USA).

**Figure 2:**
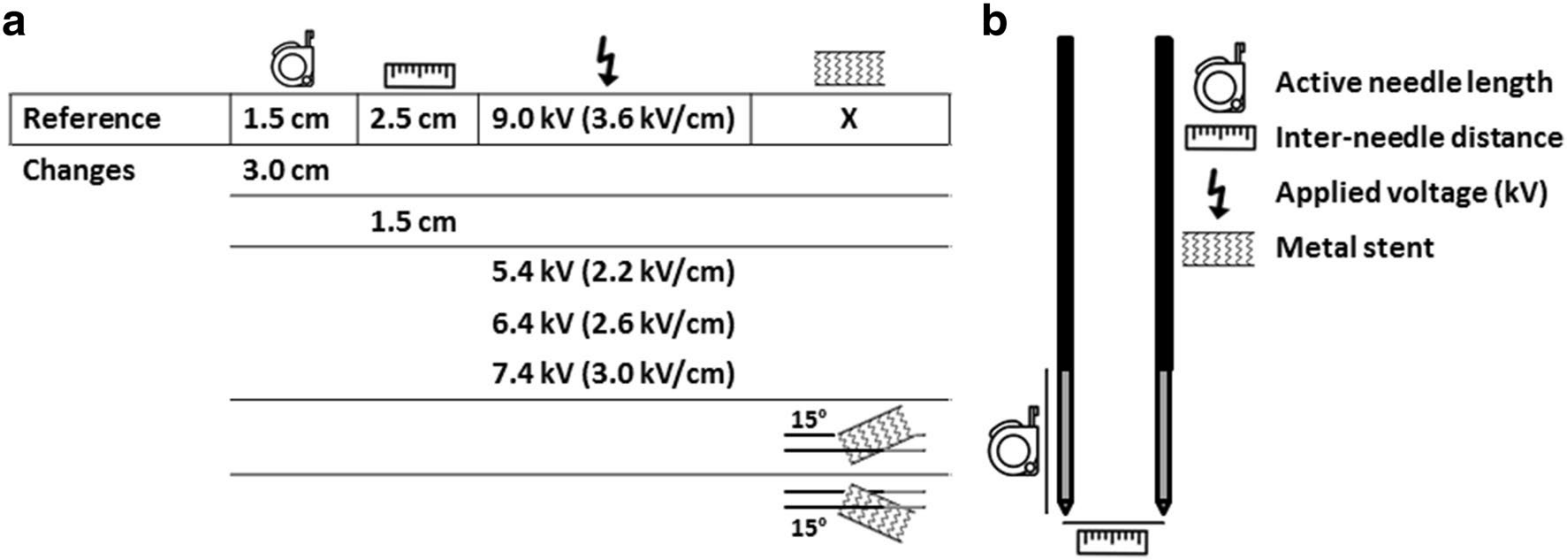
IRE parameter settings. (**a**) IRE parameter settings per experiment in comparison to the reference experiment and orientation of the metal stent when present in proximity to the needle-electrodes. (**b**) Illustration of the in the experiments varied irreversible electroporation parameters.

To evaluate the electric field line pattern, we designed a semolina in castor oil model consisting of a fixed experimental setup, containing a high-voltage generator and two needle-electrodes placed in a transparent tube filled with castor oil and semolina (Fig. 1). Semolina (dipole) aligns according to the electric field lines when a potential difference is created between the needles. This semolina pattern was illuminated using LED-lights, visually analysed and descriptively characterized in comparison to a reference experiment (Fig. 2). We demonstrate the effect of variation in IRE parameters, active needle length, inter-needle distance and applied voltage on the electric field line pattern. Furthermore, the semolina model shows that the electric field strength is the lowest halfway between the needles and we visualize the electric field disturbance due to a nearby metal stent. The redistribution of the electric field towards the stent potentially results in an incomplete tumour ablation.

A common method for analysis of the electric field distribution are computer simulations^14,17,26,28–30^. In order to determine how our semolina in castor oil model relates to these computer simulations, two-dimensional numerical models were used to predict the electric field distribution, field line pattern and electric field strength in V/cm in the centre between the needles and compared to the fundamental results provided by the semolina model. These results were supported by two-dimensional numerical simulations.

The semolina model provides fundamental insight in the electric field by a novel and intuitive visualization of electric field lines in an IRE simulation setting. The results provided by this semolina model could be used to validate and calibrate numerical simulations, which are frequently used in clinic for IRE simulation. With exception of pulse application and specific tissue properties, the effect of specific IRE parameters settings can be investigated in the semolina in castor oil model. Therefore, the model forms a comparison with IRE.

## Results

The semolina model requires relatively high voltages (up to 9.0 kV) in comparison to clinical IRE and does not provide the ability for pulse delivery since a constant static electric field is essential in visualization of electric field lines. During all experiments, the high-voltage generator measured a current of 0.00 mA, corresponding to a current at least smaller than 5 µA, which compared to a static electric field.

### Reference experiment

**Figure 3:**
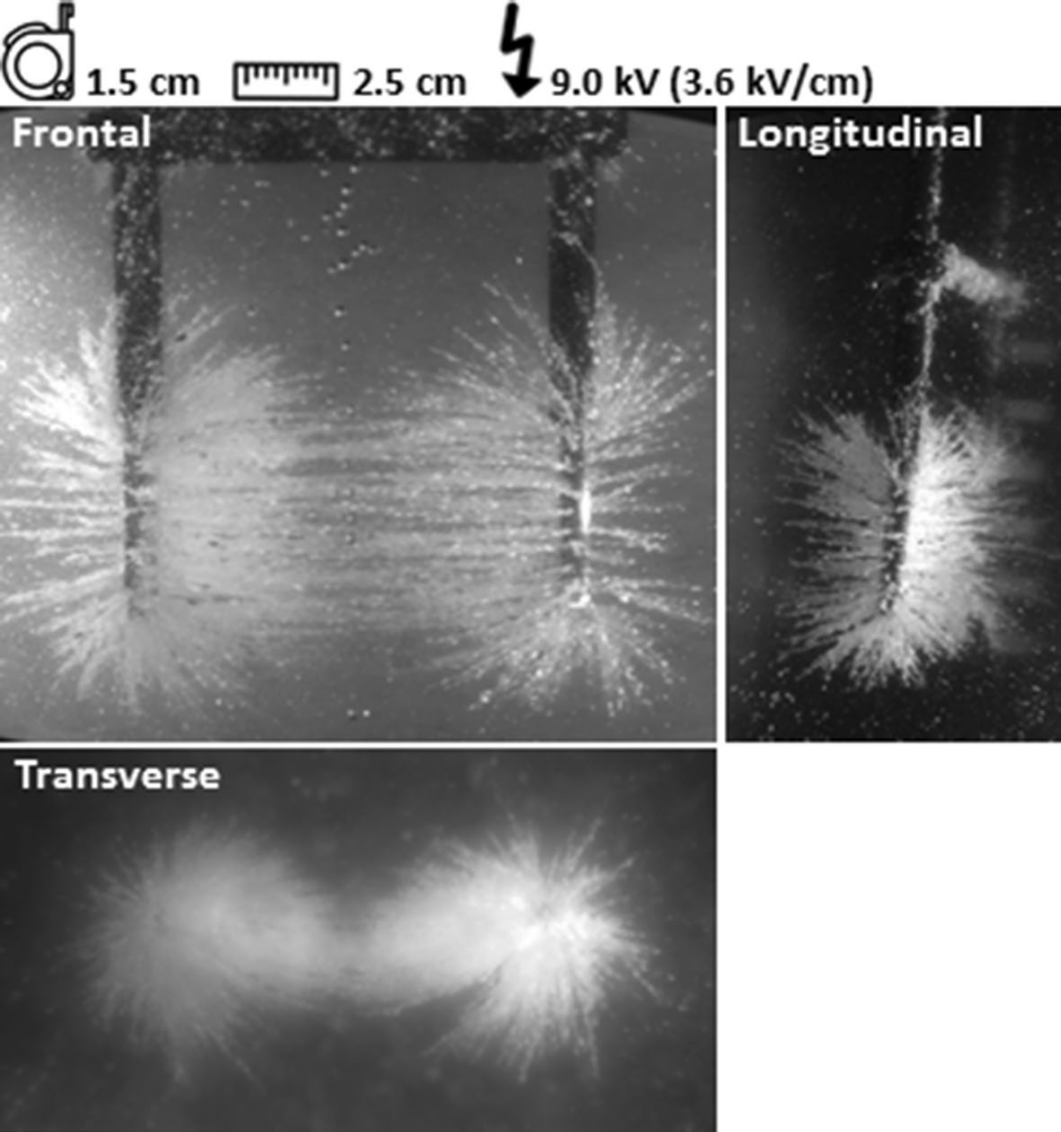
Reference experiment. The electric field line pattern is visualized in three dimensions around the needles.

The electric field line pattern was clearly visualized in the reference experiment by alignment of semolina according to the direction of the electric field (Fig. 3). Parallel electric field lines were observed in between both active needles. The spatial behavior of the electric field around the needles was best visualized in the longitudinal and transverse plane. Electric field lines fanned out in all directions around the needle tips. The highest density of electric field lines was observed in the vicinity of the needle-electrodes and decreased in all directions with increasing distance from the needles. In between the needles, downward curved lines were visualized and a relatively large semolina cloud was present directly around the needles.

### Alteration of active needle length

**Figure 4:**
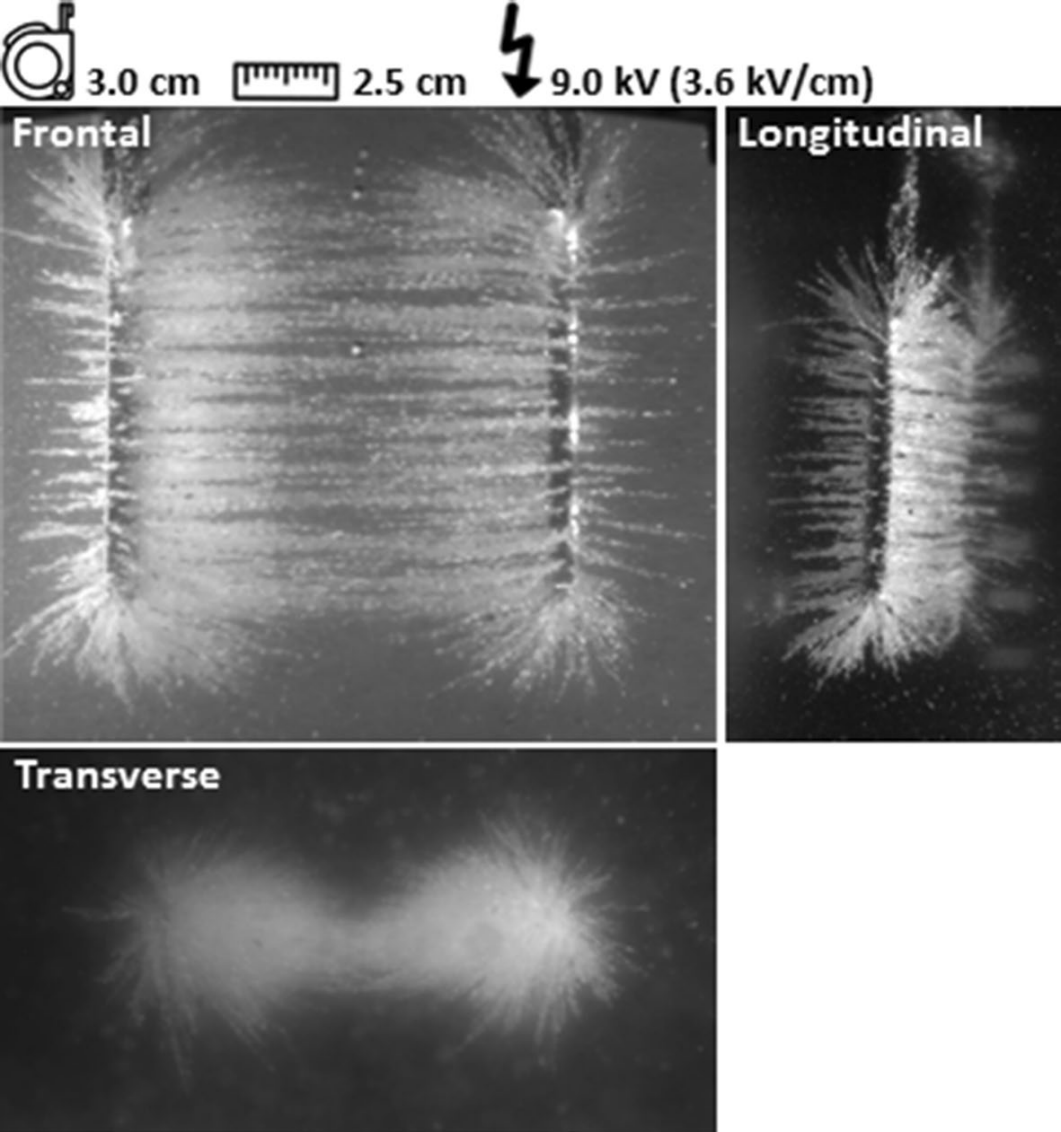
Active needle length. The Electric field line pattern is visualized for an active needle length of 3.0 cm.

The area in which electric field lines were observed increased with a larger active needle length (Fig. 4). Two phenomena were notable compared to the reference experiment: a homogeneous electric field line pattern developed between the needles, as observed in the transverse plane, and semolina was caught in a thinner slice around the needles in comparison to the reference experiment, in both the frontal and longitudinal plane.

### Alteration of inter-needle distance

**Figure 5:**
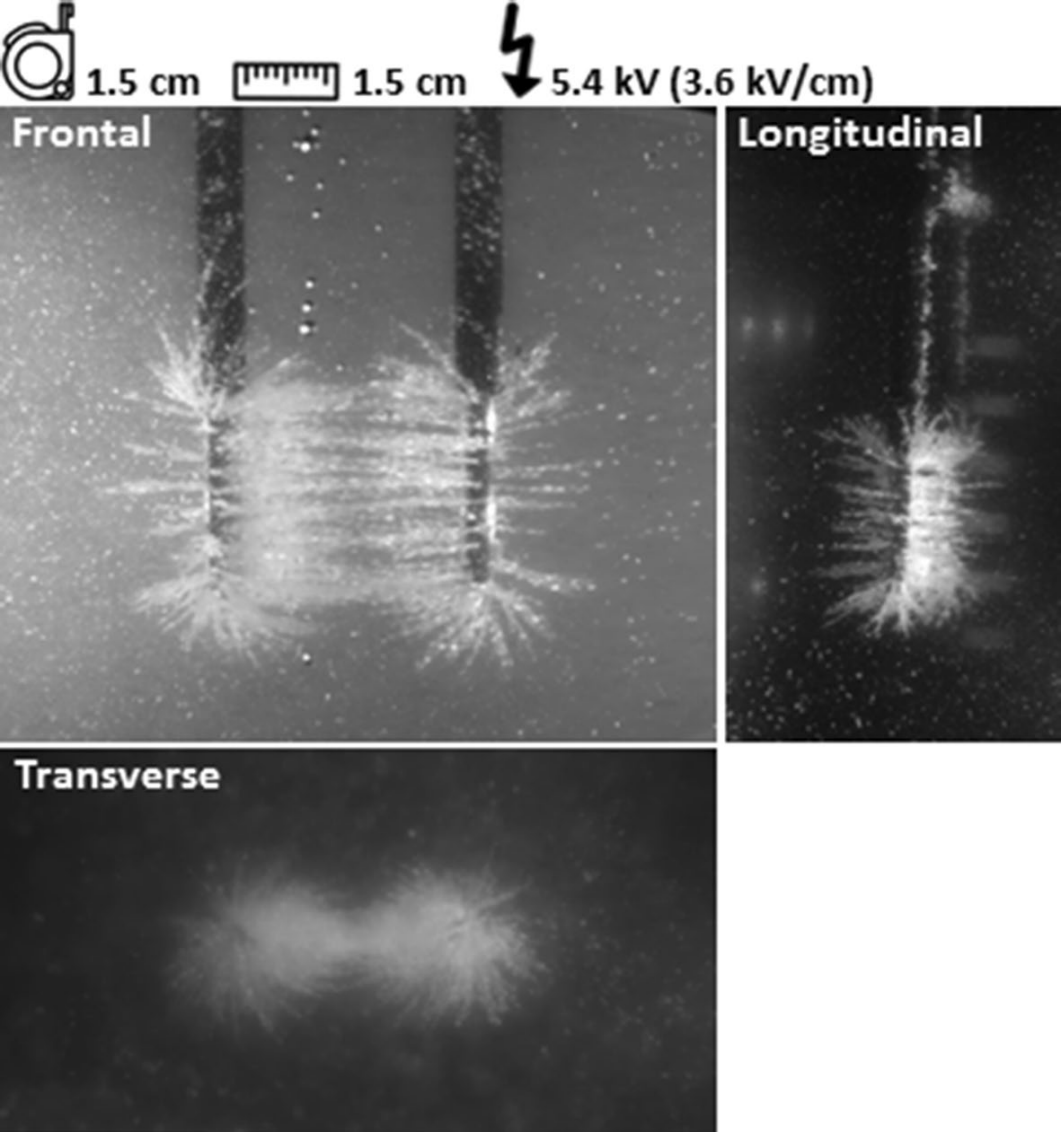
Inter-needle distance. The Electric field line pattern is visualized for an inter-needle distance of 1.5 cm.

The electric field line pattern after alteration of the inter-needle distance to 1.5 cm is visualized in Fig. 5. Electric field lines were oriented in the same way between the needles in comparison to the reference experiment. A straight line was formed between the needle tips instead of a downward curved line in the longitudinal plane. The needle closest to the camera used to capture the longitudinal image appeared to be smaller than the corresponding needle of the reference experiment because of the greater distance to the camera due to the reduction in inter-needle distance. Furthermore, semolina was captured in a thinner slice around the needles in comparison to a 2.5 cm inter-needle distance. This effect was represented by a decrease in semolina cloud directly around the needles and best visualized in the frontal and transverse plane.

### Alteration of applied voltage

**Figure 6:**
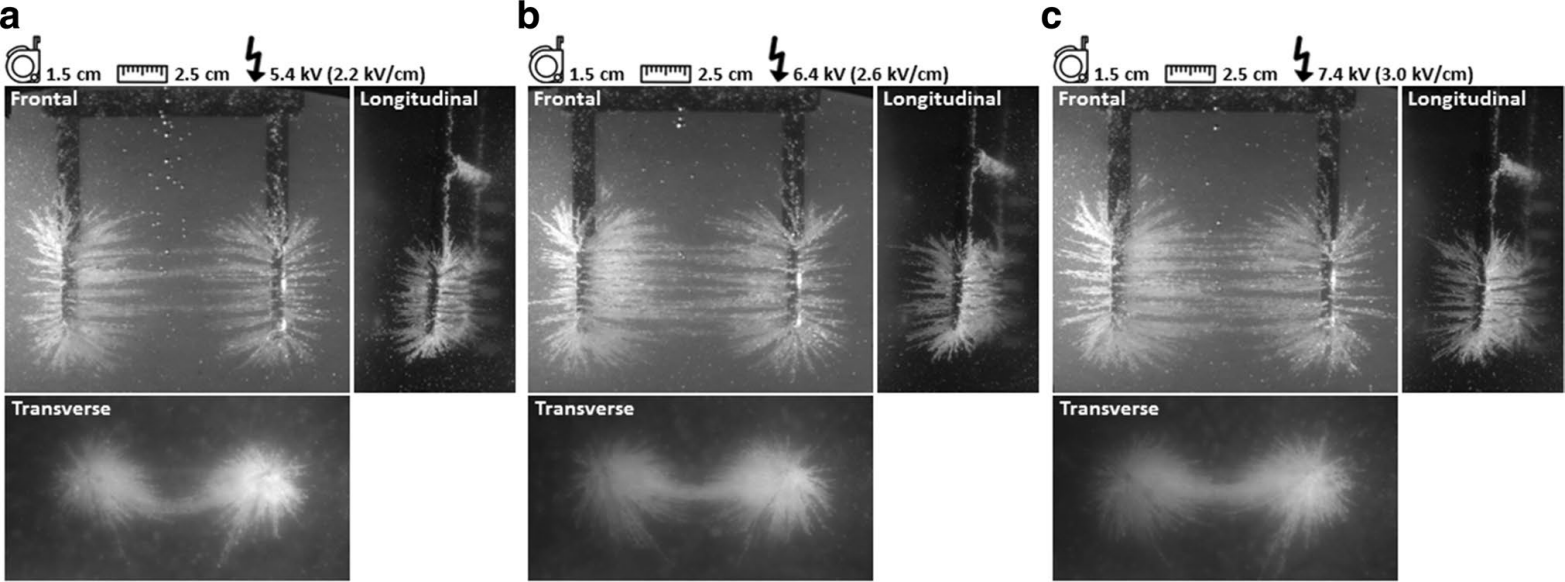
Electric field strength (kV/cm). The electric field line pattern is visualized for an inter-needle distance of 2.5 cm and applied voltage of: (**a**), 5.4 kV (2.2 kV/cm) (**b**), 6.4 kV (2.6 kV/cm) and (**c**), 7.4 kV (3.0 kV/cm).

The applied voltages of 5.4 kV, 6.4 kV and 7.4 kV in combination with an inter-needle distance of 2.5 cm corresponded to an electric field strength of 2.2 kV/cm, 2.6 kV/cm and 3.0 kV/cm respectively. Figure 6 visualizes the electric field line patterns corresponding to these electric field strengths. In comparison to the reference experiment (3.6 kV/cm (9.0 kV)), an interruption in electric field line pattern was observed halfway between the needles for all applied voltages in this part of the experiment; concerning an applied voltage of 5.4, 6.4 and 7.4 kV. There, the electric field was not strong enough to hold semolina. The interruption was less pronounced and electric field lines were better visible when the electric field strength increased. The density of electric field lines was visually increased directly around the entire active needle length and expanded in a larger area with increasing applied voltage. Finally, a downward curved line was present between the needles for an applied voltage of 5.4 and 6.4 kV (2.2 and 2.6 kV/cm), in contrast to the straight line observed for a potential difference of 7.4 kV (3.0 kV/cm).

### Proximity of a metal stent

**Figure 7:**
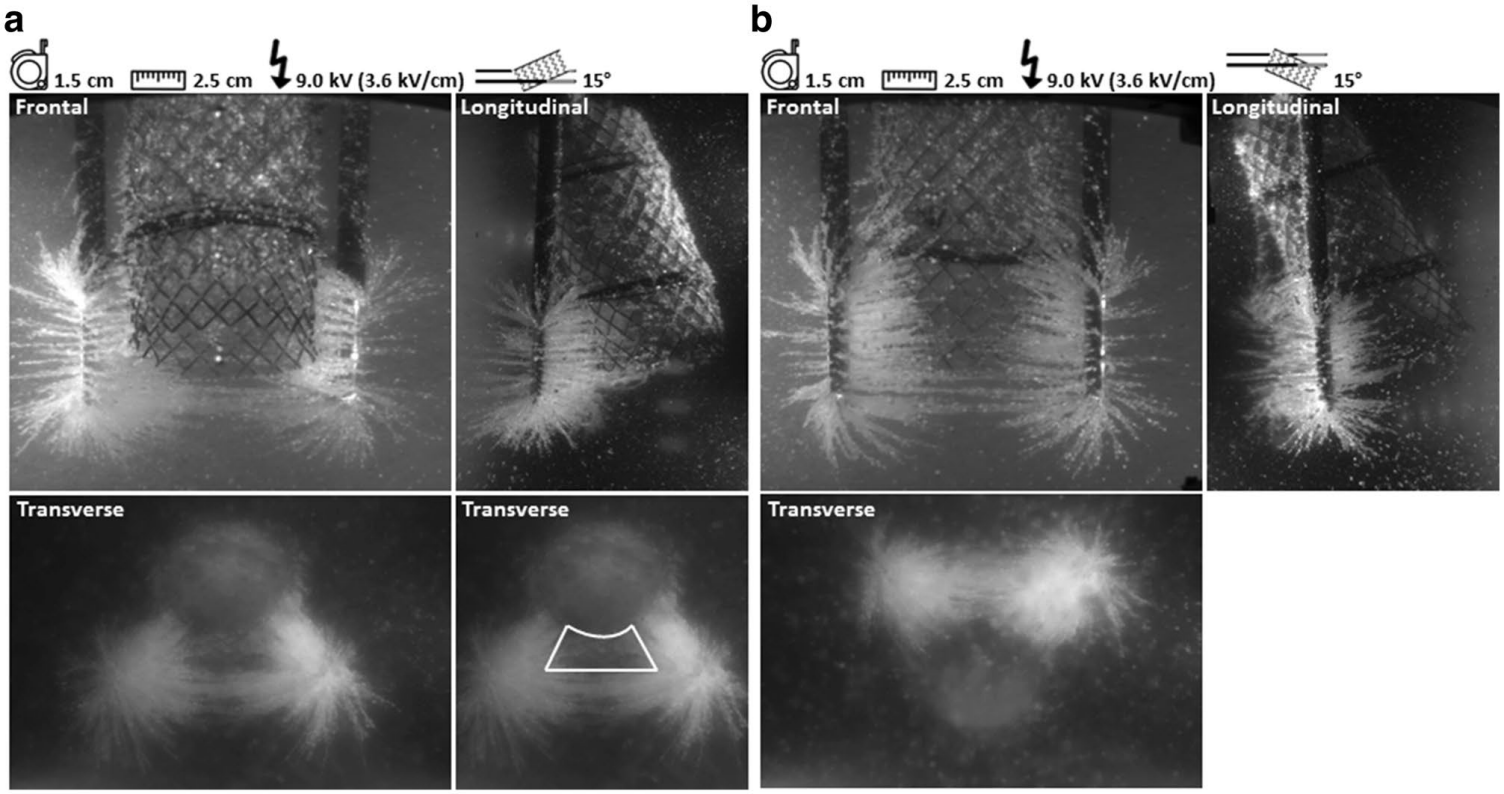
Presence of a metal stent. (**a**), A trapezoidal shaped area (demarcated white) was present in the transverse plane between the needles and the 15 degrees upwards oriented metal stent. This area demarcated a region in which no electric field lines were observed. (**b**), Electric field lines were attracted by a 15 degrees downwards oriented metal stent and resulted in an area of bare metal in the frontal plane.

The presence of a metal stent in between the needles, with a distance between the needles and stent shorter than the inter-needle distance, caused a redistribution of the electric field towards the stent (Fig. 7). Redistribution of the electric field led to a locally high density of electric field lines near the stent. The bare metal area visualized in the frontal image of Fig. 7b demonstrates that electric field lines were attracted by the metal; no semolina was aggregated beneath the stent. Notably, no electric field lines were visible inside a trapezoidal shaped area located between the needles and the stent and inside the lumen of the stent. This effect was best observed in images representing electric field lines in the transverse plane.

### Numerical electric field simulations in two dimensions

**Figure 8:**
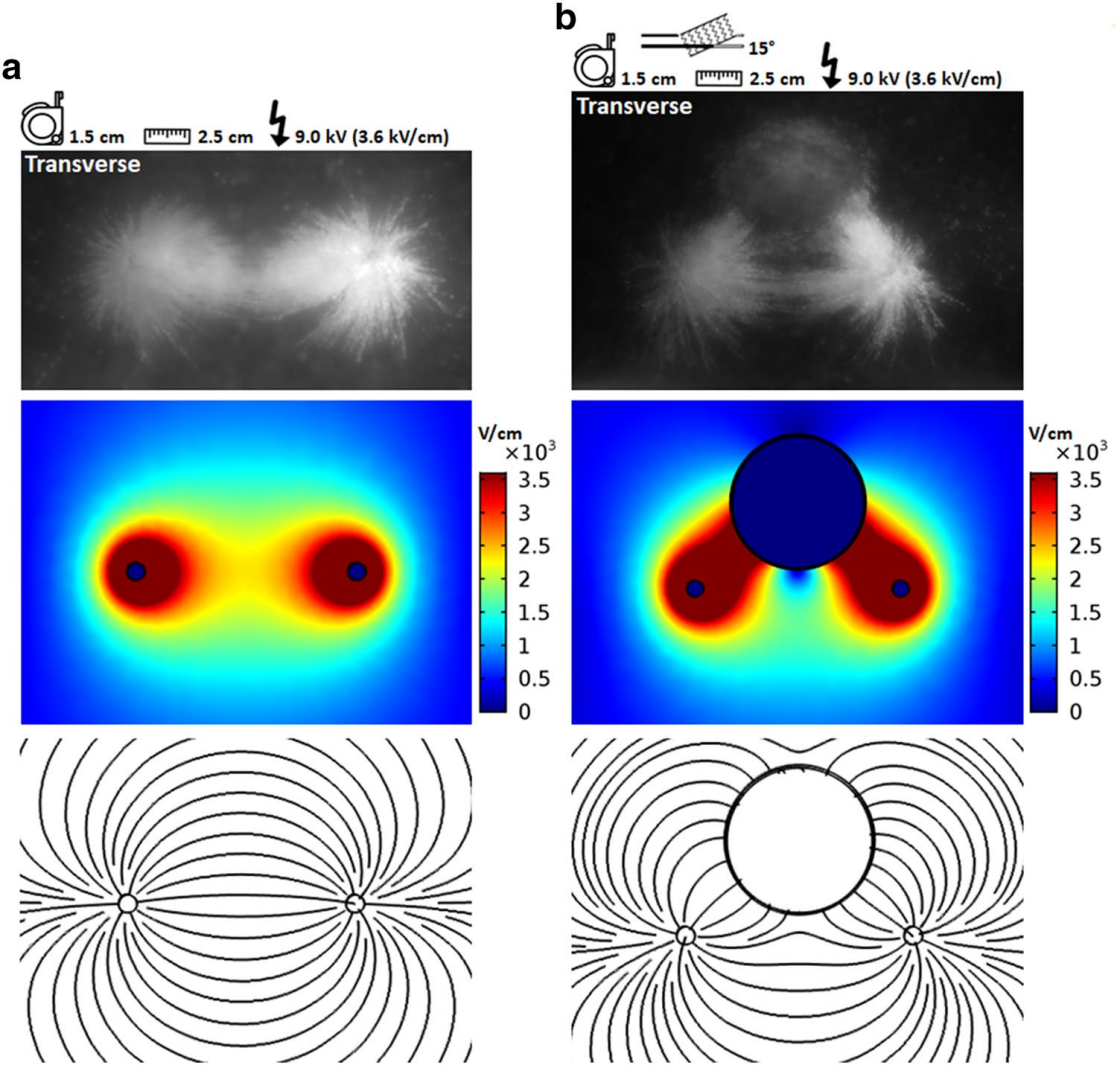
Numerical simulations of the electric field distribution and electric field line pattern in castor oil in two dimensions. Results provided by the semolina in castor oil model in comparison to the electric field distribution (V/cm) and electric field line pattern in the transverse plane (**a**), in absence of a metal stent for 9.0 kV and (**b**), in presence of a 15 degrees upward oriented metal stent for 9.0 kV. COMSOL Multiphysics (COMSOL, v5.3, www.comsol.com, Stockholm, Sweden).

Two-dimensional numerical simulations were performed to predict the electric field distribution and electric field line patterns for an inter-needle distance of 2.5 cm in presence and in absence of a metal stent (Fig. 8). Castor oil was used as medium during the numerical simulations. Findings in the semolina model corresponded well with the numerical simulation results. Red coloured regions in the electric field distribution were ranged to 3600 V/cm, representing the highest electric field strengths. The highest electric field strengths were observed directly around the needles and along the path between the needles and stent. These regions corresponded to the areas in which most semolina was caught. Figure 6 showed an interruption in electric field line pattern halfway between the needles, there the electric field strength was the lowest and conform the numerical simulations. Numerical simulations in castor oil demonstrated an electric field strength of 1405 V/cm, 1665 V/cm and 1925 V/cm in the centre between the needles for a potential difference of 5.4 kV, 6.4 kV and 7.4 kV respectively. From Fig. 6b can be observed that electric field strengths in the order of magnitude of > 1665 V/cm were required for semolina to be caught and enable visualization of the electric field lines. A similar electric field line pattern was observed between the semolina in castor oil and numerical simulation models. The electric field was absent inside the metal stent (dark blue coloured region, 0 V/cm), redistributed towards the metal and a locally high electric field strength was observed along the shortest path between the needles and stent. Lowest electric field strengths were observed in a trapezoidal shaped area between the needles and stent in both the semolina model and numerical simulations. Halfway between the needles, the electric field strength decreased by a factor of two, from 2341 to 1130 V/cm for an applied voltage of 9.0 kV, when a metal stent was placed in the ablation area. The same factor in electric field strength reduction was observed in the centre between the needles for a potential difference of 3.0 kV, the electric field strength decreased from 780 to 377 V/cm in presence of a metal stent. A comparable pattern in electric field distribution was observed for an applied voltage of 3.0 kV as well as for 9.0 kV. Except from higher electric field strengths used when 9.0 kV was applied.

## Discussion

The effect of selected IRE parameters and presence of a metal stent on the electric field line pattern were successfully visualized by the semolina model in three dimensions, supported by two-dimensional numerical simulations of the electric field distribution and field line pattern. The distribution of electric field lines around and between the needles as well as the density of electric field lines were clearly influenced by alteration of the inter-needle distance, active needle length and applied voltage. Electric field lines were not visualized in a trapezoidal shaped area adjacent to the metal stent due to disturbance of the electric field. Numerical simulations demonstrated a substantial reduction in electric field strength in the trapezoidal shaped area, in this specific needle-stent configuration a factor of two reduction. Building upon this information, areas of potential incomplete IRE ablation near a metal stent and locations where thermal effects may occur could be determined.

A metal stent leads to a substantial reduction in electric field strength in the area halfway between the needles and gives rise to areas with potentially insufficient electric field strength to induce irreversible electroporation. Numerical simulations of the 3.0 kV potential difference confirmed that the electric field strength was below the IRE threshold of 680 V/cm for an effective tumour ablation in the centre between the needles in the presence of a metal stent^29,31^. While the required electric field strength was achieved in absence of a stent. The trapezoidal shaped area, outlined by a redistribution of the aggregated semolina between the needles and stent, confirms the occurrence of such areas. This fits with the observation of vital liver tissue directly adjacent to the stent observed after IRE in in vivo porcine liver^19^. Prior animal studies and experimental research revealed that proximity of a metal stent resulted in an unpredictable asymmetrical ablation zone and potential ineffective tumour ablation, theoretically explained by attraction of the electric field^15,16,18–20^. How and to what extent the electric field is disturbed by a metal stent was not clear. This hampered the ability to predict the deformation of the ablation zone and to effectively adjust for this during IRE treatment in presence of a metal stent. Our semolina model provides insight in the occurrence of electric field disturbances. The aggregation of semolina according to the electric field can be used to visualize the areas of (in)effective ablation and identify possible locations where undesired thermal effects may occur in the presence of a metal stent. Our study visualized the redistribution of the electric field towards the metal due to its lower electrical resistance in comparison to castor oil or tissue. Redistribution led to a locally high density of electric field lines between the needles and stent and corresponded to the areas of highest electric field strength along the path between the needles and stent as observed by numerical simulations. These observations could explain the increase in current in presence of a metal stent as observed in previous studies and occurrence of thermal effects directly around the needles and along the path between the needles and stent^15,16,19^. It is important to realize that the distance between the metal stent and ablation zone or IRE electrodes in combination with the size of the metal object determines whether the electric field is significantly disturbed^14,17^. Clinicians should be aware of the reduction in electric field strength as a consequence of a metal stent near the IRE electrodes. An increase in pulse number could potentially at least partially counteract the occurrence of an ineffective tumour ablation near a metal stent^14^. However, it is a delicate balance between an effective ablation and thermal effects or damage.

The occurrence of thermal injury during a regular IRE treatment and the impact of a metal stent on the local temperature distribution and thermal injury remains controversial^4,13,16–21,23,26,32–34^. Severe complications during IRE of a pancreas tumour were presumed to be related to heating of the metal stent in a case report^13^. Mean maximum temperatures of 65.3 °C and incomplete ablation were observed for IRE in the presence of a metal clip or stent within the ablation zone in porcine liver versus 47.3 °C for absence of metal (*p* = 0.007)^16^. Multiple studies that performed temperature measurements during IRE without proximity of a metal stent near the needle-electrodes, showed divergent results, varying from a maximum temperature rise of 1.15 °C and successful ablations without thermal damage to measured peak temperatures of 79 °C with certain thermal damage^16,21,23,26,32–34^. The occurrence of thermal effects and thermal damage depend on the IRE protocol and heat transfer to surrounding tissue during ablation^10,23,26,34^. Based on the observed semolina aggregation and numerical simulations in absence of a metal stent, the area most at risk of thermal effects and damage is the area directly adjacent to the needles. All electric field lines leave and enter the needle in that small area. Our finding of a locally high density of electric field lines near the needles corresponds to a high current density and coincides well with experimental observations of temperature increase near the needles^21,22,33,35^. Potential thermal effects near the needles and incomplete tumour ablation in presence of a metal stent should be predicted and considered during IRE treatment planning to ensure an effective and safe IRE procedure.

We observed that the electric field strength and density of electric field lines were the lowest halfway between the needles in both the semolina model and numerical simulations. This observation is in accordance with the physical behaviour of an electric field between two point charges^35^. A relatively low electric field strength halfway between the needles makes it harder to reach the required IRE threshold of 680 V/cm and potentially limiting an adequate tumour ablation in this area^29,31^. The observed interruption in electric field strength in Fig. 6 is explained by the variation of electric field strength per area. The field strength is dependent on the local density of electric field lines, strength of every single electric field line present in that area and distance of that area to the needles^35^. The electric field strength should be > 1665 V/cm to visualize an uninterrupted electric field line pattern (Fig. 6). An increased active needle length and decreased inter-needle distance resulted in a decreased semolina cloud (Figs. 3, 4 and 5), associated with a decrease in electric field strength. A potential clinical consequence is ablating a thinner slice of tissue around the needles than expected. Therefore, the potential difference and subsequent electric field strength should hypothetically be increased to create the desired ablation zone. Clinicians should be aware of the consequences of adjustment in parameter settings on electric field strength, especially halfway the needles, during IRE treatment.

The visualization experiments have several strengths and limitations. A strength is that this is the first study in which the electric field lines for selected IRE settings frequently used in clinical practice have been visualized based on a unique semolina in castor oil model, completely different from the currently available simulation and experimental models. It is not possible to visualize electric field line patterns in tissue or gel phantoms. Our model is a fundamental method to investigate the effect of changes in IRE parameters on the electric field and the extent a metal stent disturbs the electric field. High quality and reproducible images were acquired during all experiments by the fixed experimental setup and adjustable LED-lights. A limitation is that the potential difference used in our experiment is about a factor of three higher than those used in the clinical setting. Higher potential differences result in a locally denser and more extensively fanned out electric field lines. This may result in an overestimation of the ablation zone size when directly related to tissue IRE. However, the pattern the electric field lines together form is unchanged, confirmed by the comparable pattern in electric field distribution for both an applied voltage of 3.0 kV and 9.0 kV. Also the use of a static electric field instead of a dynamic electric field and castor oil as medium instead of tissue are not directly comparable to the in vivo setting^15,36^. To clearly visualize electric field lines, the electric field must be static and of sufficient strength (> 1665 V/cm, Fig. 6) since semolina can only be caught when the electric field is continuously maintained over time. The electric field strength must exceed the gravitational force on semolina to visualize the electric field line pattern. The use of a static electric field is essential but makes it impossible to deliver electric pulses and vary the pulse length during the experiments. All other parameter values investigated in this study resemble clinical practice. In clinic, tissue inhomogeneities will substantially influence the shape of the ablation zone^15,28,37^. But the electric field adaptation to changes in IRE parameters in vivo will be comparable to the extent the electric field line pattern reorganizes in castor oil since a current will always follow the path of lowest resistance. Nonetheless, our semolina model still offers a method for testing the influence of combinations of IRE parameter values in specific cases.

Reliable prediction of the disturbance of the electric field in presence of a metal stent and location of thermal effects are a prerequisite for effective and safe IRE procedures. The semolina model provides an intuitive visualization of electric field lines, the fundamental physics when IRE parameter values were varied. The results provided by this semolina model can be used to validate and calibrate numerical simulations, which are frequently used in clinic for IRE simulation. The semolina model can be used potentially to provide insight in areas of effective IRE ablation and potential thermal effects, based on the observed density and presence of electric field lines. In a pre-procedural setting, a patient specific planning of the needle position with reference to the stent can be made based on the semolina model and numerical simulations to limit the disturbance of the electric field. However, experiments performed in an IRE setting in tissue (mimicking media) in combination with semolina experiments and numerical simulations are desirable to obtain a more profound insight in clinical IRE and personalized treatment.

In conclusion, we demonstrated a semolina in castor oil model to intuitively visualize the electric field line pattern, allowing insight into the effect of combinations of selected clinical IRE ablation parameters. The results provided by the semolina model were supported by two-dimensional numerical simulations. A disturbance and redistribution of the electric field was shown in combination with a substantial reduction in electric field strength in both the semolina model as well as by numerical simulations when a metal stent was placed near the needle-electrodes. Caution should be taken when performing IRE in vicinity of a metal stent, which could give rise to unpredictable heating and incomplete tumour ablation.

## Methods

### Experimental setup

The electric field line pattern was visualized in a static electric field by semolina (dipole) in castor oil (Ricinus oil k.p. Ph.Eur., De Lange, Belfeld, The Netherlands) (electric insulator)^35,38,39^. A visualization of the experimental setup is presented in Fig. 1. The experimental setup consisted of a frame built of polyoxymethylene (POM, Epratal-C), that held an optically transparent 20 cm polymethylmethacrylate (Perspex) hollow tube (Ø80/74 mm), sealed with Perspex lids. The tube was filled with ten grams of semolina, supplemented with castor oil until the tube was completely filled. Herein two rigid 21 cm 316 Stainless Steel needles (Ø2 mm), with sharp needle tip, were placed. These needles were placed in parallel at an inter-needle distance of 1.5 or 2.5 cm around the centre point of the lid, dependent on the experiment. Each needle-electrode pair was placed in the horizontalplane. Both needle-electrodes were electrically insulated by a heat shrinkable sleeve, except for the active needle length. A high-voltage generator (MPL 500–10,000, FuG Elektronik GmbH, Schechen, Germany) was used to create a static electric field between the needles. To be able to create the desired electric field strength, the left needle was internally kept close to the ground potential and the right needle was positively charged to the specific voltage. The current in the circuitry was measured continuously by the high-voltage generator. Despite the presence of a static electric field, the current was for safety reasons limited to 0.50 mA.

### IRE parameter values

The effect of variation in inter-needle distance, active needle length and applied voltage (kV) on the electric field line pattern were assessed (Fig. 2). As reference, the electric field line pattern obtained with an inter-needle distance of 2.5 cm, 1.5 cm active needle length and 9.0 kV potential difference (3.6 kV/cm electric field strength) was used. The active needle length and inter-needle distance values chosen as reference are commonly used to treat locally advanced pancreatic cancer. In the experiments, the electric field strength (kV/cm) was calculated by dividing the applied voltage by the inter-needle distance, rounded to one decimal place based on the precision of the measurement system. The effect of each IRE parameter was visualized by varying that specific parameter of interest with respect to the reference experiment while all other parameters remained unchanged (Fig. 2). To investigate the effect of a metal stent, a customized self-expandable nitinol gastrointestinal metal stent, Ø18 mm and 45 mm length, (Epic, Boston Scientific, Marlborough, Massachusetts, US) was placed in between both active needle lengths in two configurations, oriented upwards for fifteen degrees as well as downwards in the same angle. The stent itself was electrically insulated from the needles and the shortest distance between the active needle length and stent was 1.0 cm. This needle-stent configuration happens sometimes in clinical practice. Visualization of the electric field distribution for an upward and downward oriented metal stent was performed to investigate the dependence of the electric field line pattern on the stent configuration.

### Electric field visualization

Electric field lines were visualized by aggregation of semolina where the electric field strength in combination with the force exerted by the viscous castor oil exceeded the gravitational force on semolina. Three camera body (Basler Ace 2 acA1920-40um mono, Basler, Ahrensburg, Germany)—lens combinations (Pentax 12 mm f/1.4 c-mount H1214-M) were used to visualize the development of electric field lines in the frontal, longitudinal and transverse plane of the needles (Fig. 1). Each camera was focused on the needle-electrodes in the corresponding image plane (frontal plane: focused on the plane crossing through both needles, longitudinal plane: focused in the plane in between both needles, transverse plane: focused on the needle tips). Semolina patterns were illuminated by four 5 cm LED-lights (Cool white 6000 K, 1950 lm/meter). A suitable combination of LED-lights and light intensity of every single LED-light was determined manually for every image plane using ‘Processing’ software (Version 3.5.3, Processing foundation). The cameras and LED-lights were externally triggered simultaneously by an Arduino board (Arduino Mega2560 Rev3, Arduino.cc) in combination with open-source Arduino IDE software (Version 1.8.11, Arduino, Arduino.cc) for image capturing by the Pylon viewer software (Version 5.0.12, Basler, Ahrensburg, Germany). As a last step before an experiment started, the semolina-castor oil substance was mixed by shaking the tube for five minutes and the tube was placed horizontally in the experimental setup. From the moment the potential difference was created between the needles, a waiting time of thirty minutes was used to ensure semolina which did not contribute to the formation of electric field lines, had assembled at the bottom of the tube. Images were captured by all cameras simultaneously with a frame rate of 1 image per second from the moment the potential difference was created between the needles. The images obtained after the first thirty minutes of an experiment, compared to a static electric field in three dimensions, were used for image analysis.

### Numerical simulations

**Figure 9:**
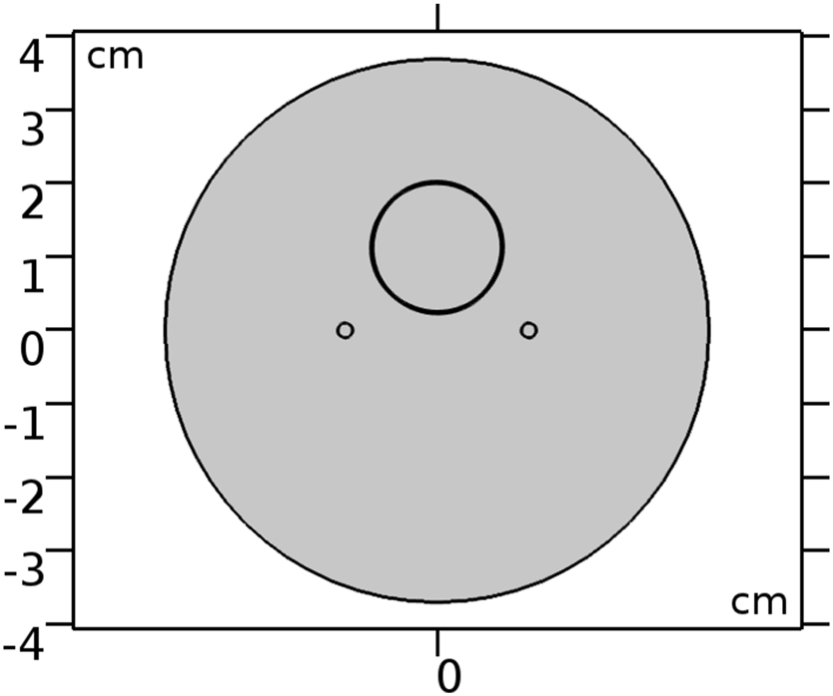
Two-dimensional geometry numerical model. Two stainless steel 316 needle-electrodes (2.5 cm inter-needle distance) and a nitinol metal stent (0.25 mm strut thickness) were placed in a tube filled with castor oil. COMSOL Multiphysics (COMSOL, v5.3, www.comsol.com, Stockholm, Sweden).

A two-dimensional Perspex tube (Ø74 mm) with two stainless steel needles (Ø2 mm, separated by 2.5 cm) and a nitinol metal stent (Ø18 mm, 0.25 mm strut thickness) inside was built to perform numerical simulations in castor oil (Fig. 9). COMSOL Multiphysics (COMSOL, v5.3, Stockholm, Sweden) finite element software was used to solve the Laplace Eq. (1)^30^:

1 $$\nabla \bullet (\sigma \nabla \varphi )=0$$

where $\sigma$ is the electrical conductivity of the medium and $\varphi$ the applied electric potential. The boundary conditions of the electrodes were given by $\varphi$ = 0 (ground) and $\varphi$ = voltage applied during the experiments. The exterior boundary of castor oil was treated as electrically insulative. A floating potential was added to the outer boundary of the stent and the inner boundary was electrically insulated.

Numerical simulations were used to predict the electric field distribution (V/cm) and electric field line pattern for an inter-needle distance of 2.5 cm and applied voltage of 3.0 kV and 9.0 kV in presence as well as in absence of a nitinol metal stent. The electric field strength was also evaluated halfway between the needles in point (0,0) for applied potential differences of 3.0, 5.4, 6.4, 7.4 and 9.0 kV in absence of a metal stent and for both 3.0 kV and 9.0 kV in presence of a metal stent. Table 1 summarized the material properties used in the numerical model.

**Table 1:** Properties used within the numerical model.

Material	Property	Value	Unit	References
Castor oil	Electrical conductivity	2 × 10^–12^ (40 °C)	S/m	^40^
	Relative permittivity	4.35 (20 °C)	–	^41^
Stainless steel	Electrical conductivity	1.30 × 10^6^ (20 °C)	S/m	^42^
	Relative permittivity	1	–	
Nitinol	Electrical conductivity	1.32 × 10^6^ (20 °C)	S/m	^43^
	Relative permittivity	1	–	

### Image analysis

The raw images were cropped uniformly to the same area in the centre of the image, the image ratio was unchanged. The obtained semolina pattern images were visually analyzed and characterized descriptively. Results provided by the two-dimensional numerical models were compared qualitatively to the results provided by the semolina in castor oil model.

## Data Availability

The data that support the findings of this study are available from the corresponding author upon reasonable request.
